# Identification of Potential Prognostic Competing Triplets in High-Grade Serous Ovarian Cancer

**DOI:** 10.3389/fgene.2020.607722

**Published:** 2021-01-13

**Authors:** Jian Zhao, Xiaofeng Song, Tianyi Xu, Qichang Yang, Jingjing Liu, Bin Jiang, Jing Wu

**Affiliations:** ^1^Department of Biomedical Engineering, Nanjing University of Aeronautics and Astronautics, Nanjing, China; ^2^College of Automation Engineering, Nanjing University of Aeronautics and Astronautics, Nanjing, China; ^3^School of Biomedical Engineering and Informatics, Nanjing Medical University, Nanjing, China

**Keywords:** lncRNA, ceRNA, competing triplet, LncMiM, ovarian cancer

## Abstract

Increasing lncRNA-associated competing triplets were found to play important roles in cancers. With the accumulation of high-throughput sequencing data in public databases, the size of available tumor samples is becoming larger and larger, which introduces new challenges to identify competing triplets. Here, we developed a novel method, called LncMiM, to detect the lncRNA–miRNA–mRNA competing triplets in ovarian cancer with tumor samples from the TCGA database. In LncMiM, non-linear correlation analysis is used to cover the problem of weak correlations between miRNA–target pairs, which is mainly due to the difference in the magnitude of the expression level. In addition, besides the miRNA, the impact of lncRNA and mRNA on the interactions in triplets is also considered to improve the identification sensitivity of LncMiM without reducing its accuracy. By using LncMiM, a total of 847 lncRNA-associated competing triplets were found. All the competing triplets form a miRNA–lncRNA pair centered regulatory network, in which ZFAS1, SNHG29, GAS5, AC112491.1, and AC099850.4 are the top five lncRNAs with most connections. The results of biological process and KEGG pathway enrichment analysis indicates that the competing triplets are mainly associated with cell division, cell proliferation, cell cycle, oocyte meiosis, oxidative phosphorylation, ribosome, and p53 signaling pathway. Through survival analysis, 107 potential prognostic biomarkers are found in the competing triplets, including FGD5-AS1, HCP5, HMGN4, TACC3, and so on. LncMiM is available at https://github.com/xiaofengsong/LncMiM.

## Introduction

Non-coding RNAs (ncRNAs) were once considered as junk RNAs; however, evidence has increasingly shown that ncRNAs can perform diverse functions (Slack and Chinnaiyan, 2019; Yao et al., 2019; Chen et al., 2020; Nair et al., 2020). Among ncRNAs, the most intensively studied subclass are microRNAs (miRNAs, usually 19–24 nucleotides long), which can regulate gene expression posttranscriptionally by destabilizing target mRNAs via the RNA-induced silencing complex (RISC) (Bartel, 2009; Gebert and MacRae, 2019). The miRNA-based regulation has been reported to be involved in many pathologies including cancer (Peng and Croce, 2016; Dhawan et al., 2018; Huang et al., 2019). By contrast, the other class of abundant ncRNAs, lncRNAs (>200 nucleotides long), are still less understood, although much larger numbers of lncRNAs have been identified using high-throughput sequencing techniques in recent years (Fang et al., 2018; Frankish et al., 2019; Volders et al., 2019). Nevertheless, the existing well-characterized lncRNAs have demonstrated their important roles in various critical biological processes, such as chromatin remodeling, genomic splicing, cell proliferation, and cell differentiation (Fatica and Bozzoni, 2014; Han and Chang, 2015; Romero-Barrios et al., 2018; Rossi et al., 2019; Yao et al., 2019). In addition, dysregulation of lncRNAs is implicated in various human diseases (Schmitt and Chang, 2016; Bao et al., 2019; Gao et al., 2019).

Recent studies prove that lncRNAs participate in the posttranscriptional regulation by acting as competing endogenous RNAs (ceRNAs) (Song et al., 2017; He et al., 2019). The lncRNAs that share miRNA response elements (MREs) with mRNAs can compete for miRNA binding, thereby alleviating the inhibitory effect of miRNAs on their mRNA targets. To date, considerable lncRNA-associated competing triplets (lncRNA–miRNA–mRNA) have been reported to be involved in cancer progression (Du et al., 2016; Cong et al., 2019; Wang et al., 2019). For example, the lncRNA *MEG3* functions as a ceRNA of oncogenic miR-181 to regulate gastric cancer progression (Peng et al., 2015, 3). The lncRNA *UCA1* upregulates the expression of *ERBB4* through competitively “sponging” miR-193a−3p and functions as an oncogene in non-small cell lung cancer (NSCLC) (Nie et al., 2016, 1). The *XIST*/miR-92b/*Smad7* triplet is found to play an important role in the progression of hepatocellular carcinoma (Zhuang et al., 2016). Hence, lncRNA associated competing triplets attract more and more attention in cancer research.

At present, several computational methods have been proposed for identifying competing triplets (Le et al., 2017; Hornakova et al., 2018). In general, people usually use linear correlations between gene–gene and/or gene–miRNA pairs to identify ceRNA triplets, since it requires a small sample size and fewer computations (Wang et al., 2015). However, the linear correlation-based methods do not measure the impact of the miRNA on the gene–gene interaction within triplets, resulting in reduced credibility of competing triplet identification results. In order to overcome this problem, several methods based on partial correlation (PC) or conditional mutual information (CMI) have been developed. Among them, two typical methods are often employed: sensitivity correlation and HERMES (Sumazin et al., 2011; Paci et al., 2014). Sensitivity correlation calculates the difference between linear correlation and partial correlation for ceRNA pairs, while HERMES calculates the difference in mutual information for each gene–gene interaction between high and low miRNA expression levels. Despite the constant increase in available methods (Wen et al., 2020), identification of competing triplets through utilizing RNA-seq and miRNA-seq data remains a challenging issue.

With the widespread application of high-throughput sequencing technology, a great deal of data has been accumulated in public databases (Lonsdale et al., 2013; Weinstein et al., 2013). The increasing data lead to more competing triplets identified by the existing methods (Wang et al., 2019); however, they also introduce some new problems needed to be solved. First, the bigger the data size, the fewer the number of linear correlated miRNA–gene pairs we could find. It seems that the relationship between the expression patterns of miRNA and its target gene is not a linear correlation as assumed by the existing methods. Second, it is noted that competing gene–gene interactions may be regulated by several miRNAs, and thus, the increased data size would make it harder to evaluate the impact of the miRNA on gene–gene interactions by using PC and CMI. In addition, besides the impact of the miRNA on gene pairs, the influence of the gene on the relationship between miRNA and other target genes should be also considered.

Here, for large data sets, we present a powerful method, named LncMiM, to identify lncRNA-associated competing triplets with a new framework addressing the above issues. From the large scale of gene and miRNA expression profiles derived from the TCGA database, 847 competing triplets were identified by using LncMiM, while only a few triplets were identified as competing ones by linear correlation-based methods. The enrichment analysis shows that they are mainly involved in cell proliferation process, cell division process, cell cycle, and ribosome pathways. Among them, 18 competing triplets were found to be associated with prognosis in high-grade serous ovarian cancer. Our method will help in identifying more lncRNA-associated competing triplets in cancer and may contribute to reveal the potential post-transcriptional regulatory mechanism of lncRNAs.

## Materials and Methods

### Data Collection and Pre-processing

**Figure 1:**
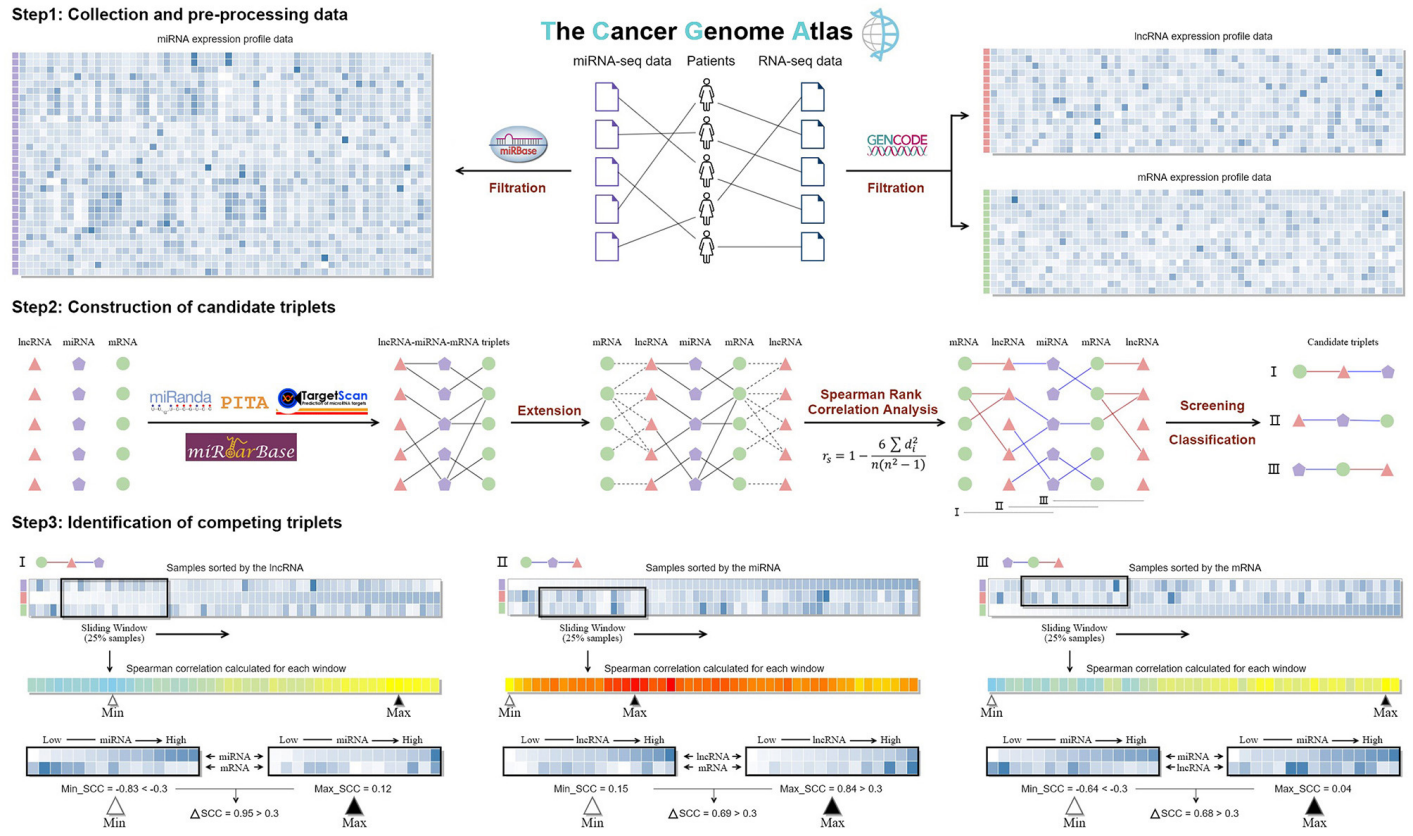
The workflow of LncMiM.

As shown in Figure 1, paired RNA-seq and miRNA-seq data of ovarian cancer (379 samples from 373 patients) are downloaded from the Cancer Genome Atlas (TCGA) (Weinstein et al., 2013). The RNA-seq data type is “Gene Expression Quantification,” and its workflow type is “HTSeq-FPKM.” The miRNA-seq data type is “Isoform Expression Quantification,” and its workflow type is “BCGSC miRNA Profiling.” The RPM (reads per million mapped reads) value was used to evaluate the expression level of miRNAs. For different samples from the same patient, we merged them by calculating the mean FPKM or RPM value for each lncRNA, mRNA, and miRNA. Finally, we got 376 samples with both the RNA-seq data and miRNA-seq data.

The annotation files of the protein-coding transcripts and the long non-coding transcripts were downloaded from the GENCODE (*version 33*) database (Harrow et al., 2012). With the transcript annotation, we extracted the mRNA expression data and the lncRNA expression data from the RNA-seq data, and the mRNAs without 3′ UTR annotation were abandoned. Human miRNA sequences and annotation were downloaded from the miRBase (*release 22.1*) database (Kozomara et al., 2019), and the seed and mature sequences of miRNAs in the miRNA-seq data were both extracted. In order to reduce the computation burden and avoid false-positive identification, we filtered out all the lower expressed RNA (mRNA, lncRNA, and miRNA) based on an artificial criterion. The remaining expressed RNAs need to be satisfied with the following conditions: (a) RNA's expressed value should be >0 in more than 75% of the 376 samples; (b) RNA's expressed value should be >5 in more than 25% of the samples; and (c) the expression variation across samples (log2IQR) should be >0.58. As a result, the expression data of 8,076 mRNAs, 225 lncRNAs, and 387 miRNAs were used for further analysis.

### Construction of Candidate Triplets

TargetScan, PITA, and miRanda are three commonly used methods to predict miRNA–target interactions (Figure 1). Due to their distinct miRNA-target predicting strategies, these methods are exclusive to any single one alone (Chiu et al., 2015). Thus, TargetScan (*version 7.2*) (Agarwal et al., 2015), PITA (*version 6*) (Kertesz et al., 2007), and miRanda (*v3.3a*) (Miranda et al., 2006) were all applied to predict miRNA–target genes. The parameters of TargetScan and PITA were set to the default values, while the score threshold of miRanda was set to 120 to get a larger miRNA–target gene pool. In addition, the experimentally validated miRNA–target interactions derived from the miRTarBase database (*release 8.0*) were also added into the miRNA–target gene dataset (Huang et al., 2020).

The lncRNA–miRNA–mRNA triplets were constructed based on the interaction relationship of miRNA–lncRNA and miRNA–mRNA; then the lncRNA and mRNA in each triplet were extracted as lncRNA–mRNA pairs. The Spearman's rank correlation coefficient (SCC) was calculated for the miRNA–lncRNA, miRNA–mRNA, and lncRNA–mRNA pairs to evaluate the regulatory relationships between miRNA, mRNA, and lncRNA in each triplet. Through a rigid screening, only 0.1% pairs were remained as functional interactions, and the cutoff values for the miRNA–lncRNA, miRNA–mRNA, and lncRNA–mRNA pairs are −0.305, −0.311, and 0.520, respectively. Based on the types of remaining interactions, candidate triplets are grouped into three classes: I, “lncRNA-centered” triplets with miRNA–lncRNA and lncRNA–mRNA interactions; II, “miRNA-centered” triplets with lncRNA–miRNA and miRNA–mRNA interactions; and III, “mRNA-centered” triplets with miRNA–mRNA and mRNA–lncRNA interactions.

### Workflow of LncMiM for Identifying Competing Triplets

For identifying competing triplets from the three types of candidate triplets, specific workflows were respectively built to evaluate the centered miRNA, lncRNA, and mRNA on the relationship between the other RNAs (Figure 1). In each workflow, samples were firstly sorted in an ascending order based on the expression of the centered RNA in the candidate triplet. The SCC of the other RNAs was calculated on the samples within the sliding window, whose size is set to 94 (25% of the total samples) and step is set to 1. And then, the maximum and minimum SCCs were calculated. Based on the type of candidate triplets, different filtering criteria were set to identify competing triplets. For the “lncRNA-centered” and “mRNA-centered” triplets, their minimum SCC should be < −0.311 and −0.305, respectively. For the “miRNA-centered” triplets, their maximum SCC should be more than 0.520. In addition, the difference between the maximum and minimum SCC should be >0.300. Finally, all the candidate triplets meeting their corresponding filtering criteria were identified as competing triplets.

In addition, to assess the statistical significance of the correlation coefficient difference (ΔCor), a series of null hypotheses were tested by measuring the ΔCor distribution over random conditions. That is, for each candidate triplet, two non-overlapping sample subsets were randomly chosen from the complete dataset, rather than based on the expression of miRNA, and then the correlation coefficient and ΔCor were calculated for these two random sample subsets. This process was repeated 100 times. The *p*-value is defined as the fraction of ΔCor in random condition that was larger than that on the specified conditions mentioned above; *p*-values were Bonferroni-corrected for the total number of candidate triplets. The triplets with adjusted *p*-values < 0.01 are statistically significant.

### Functional and Survival Analysis of the Competing Triplet

With the competing triplets, the integrative regulatory network was built and visualized by Cytoscape (Shannon et al., 2003). The size of the node and the width of the line are determined by the number of competing triplets containing them. The circular layout was produced by using the yFiles layout Algorithms. DAVID 6.8 (https://david.ncifcrf.gov) was used to perform the enrichment analysis of biological processes and KEGG pathways (Huang et al., 2009). For the enriched biological process terms, their adjusted *p*-values should be < 0.05.

The clinical profiles of 373 patients with high-grade serous ovarian cancer were downloaded from the TCGA database. The patients' ID, age at initial pathologic diagnosis, vital status, days to death, days to last follow-up, neoplasm histologic grade, and clinical stage were extracted from the clinical profiles. Based on data integrity, 369 patients' clinical data were screened out for the following survival analysis. The days to death together with the days to last follow-up make up the overall survival time of patients. Both the single variate and multivariate survival analyses used the Cox proportional hazards (PH) regression. In addition, to investigate the impact of specific genes on the survival time, patients were classified into different groups through four ways based on their expression levels. The survival analysis and visualization were performed by using the “survminer” R package.

## Results

### Investigation of the Expression Relationship Between miRNA and Target Gene

**Figure 2:**
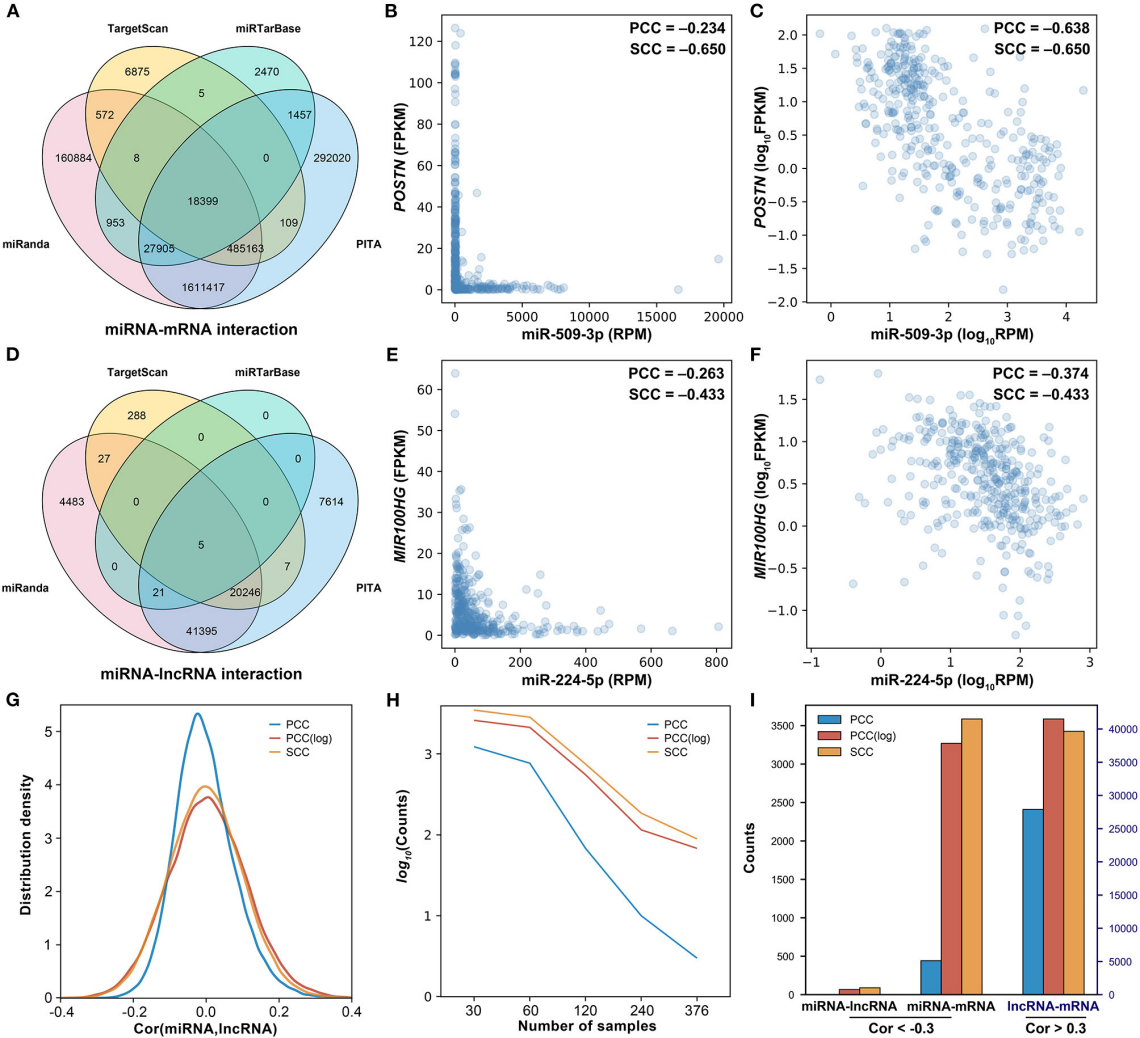
Investigation of the correlation between miRNA and the target. **(A)** The Venn diagram of miRNA–mRNA pairs. **(B)** The scatter plot of miR-509-3p and *POSTN*. **(C)** The scatter plot of miR-509-3p and *POSTN* after logarithmic transformation. **(D)** The Venn diagram of miRNA–lncRNA pairs. **(E)** The scatter plot of miR-224-5p and *MIR100HG*. **(F)** The scatter plot of miR-224-5p and *MIR100HG* after logarithmic transformation. **(H)** The density distribution of the correlation between miRNA and lncRNA. PCC, Pearson correlation; PCC(log), Pearson correlation after logarithmic transformation; SCC, Spearman correlation. **(G)** The influence of the sample size on the identification of the negatively correlated miRNA–lncRNA pairs. Negative correlation: cor(miRNA,lncRNA) < −0.3, *p*-value < 0.05. **(I)** The counts of correlated miRNA–lncRNA, miRNA–mRNA, and lncRNA–mRNA calculated on 376 ovarian cancer samples.

In general, miRNAs are assumed to be linearly correlated with their target genes. Thus, the Pearson correlation coefficient (PCC) was initially used to identify negatively correlated miRNA–mRNA and miRNA–lncRNA pairs. With the threshold of −0.30, from the 74,086 miRNA–lncRNA pairs and 2,608,237 miRNA–mRNA pairs (Figures 2A,E), only 3 miRNA–lncRNA pairs and 443 miRNA–mRNA pairs were found to be negatively correlated, which are far less than expected. As shown in Figure 2B, there is a negative regulatory relationship between miR-509-3p and *POSTN*, but the PCC is only −0.234. Similarly, miR-224-5p is also shown to be negatively correlated with *MIR100HG*; their PCC is −0.263 (Figure 2E). If the expression values were normalized by a logarithmic transformation, however, the PCCs of miR-509-3p–*POSTN* and miR-224-5p–*MIR100HG* change to −0.638 and −0.374, respectively (Figures 2C,F). As shown in Figures 2G,I, after the logarithmic transformation, more negatively correlated miRNA–target gene pairs were detected. In addition, with the increase in the sample size, the number of negatively correlated miRNA–lncRNA pairs (PCC < −0.3, *P*-value < 0.05) significantly decreases (Figure 2H). These results implied that PCC is not appropriate for the evaluation of the regulatory relationship between miRNA and target gene, especially for large sample data.

Here, we assumed that the relationship between miRNA and the target is not linear. As shown in Figures 2B,C,E,F, as compared with the PCC, the SCC is more accurate for assessing the relationship between miRNA and the target. In addition, the SCC is less affected by the sample size (Figure 2H) and can detect more negatively correlated miRNA–target gene pairs (Figure 2I). Thus, the SCC was used to screen negatively correlated miRNA–target pairs. From the 74,086 miRNA–lncRNA pairs and 2,608,237 miRNA–mRNA pairs, only 0.1% were respectively screened out as the negatively correlated miRNA–target pairs. A total of 72 negatively correlated miRNA–lncRNA pairs and 2,608 negatively correlated miRNA–mRNA pairs were selected, respectively, with the thresholds −0.311 and −0.305. Besides the miRNA–target pairs, with threshold 0.52, 1,806 positively correlated mRNA–lncRNA pairs were screened out from 1,816,605 candidate mRNA–lncRNA pairs.

### Investigation of the Impact on Pairwise Interaction by the Other One in Triplets

**Figure 3:**
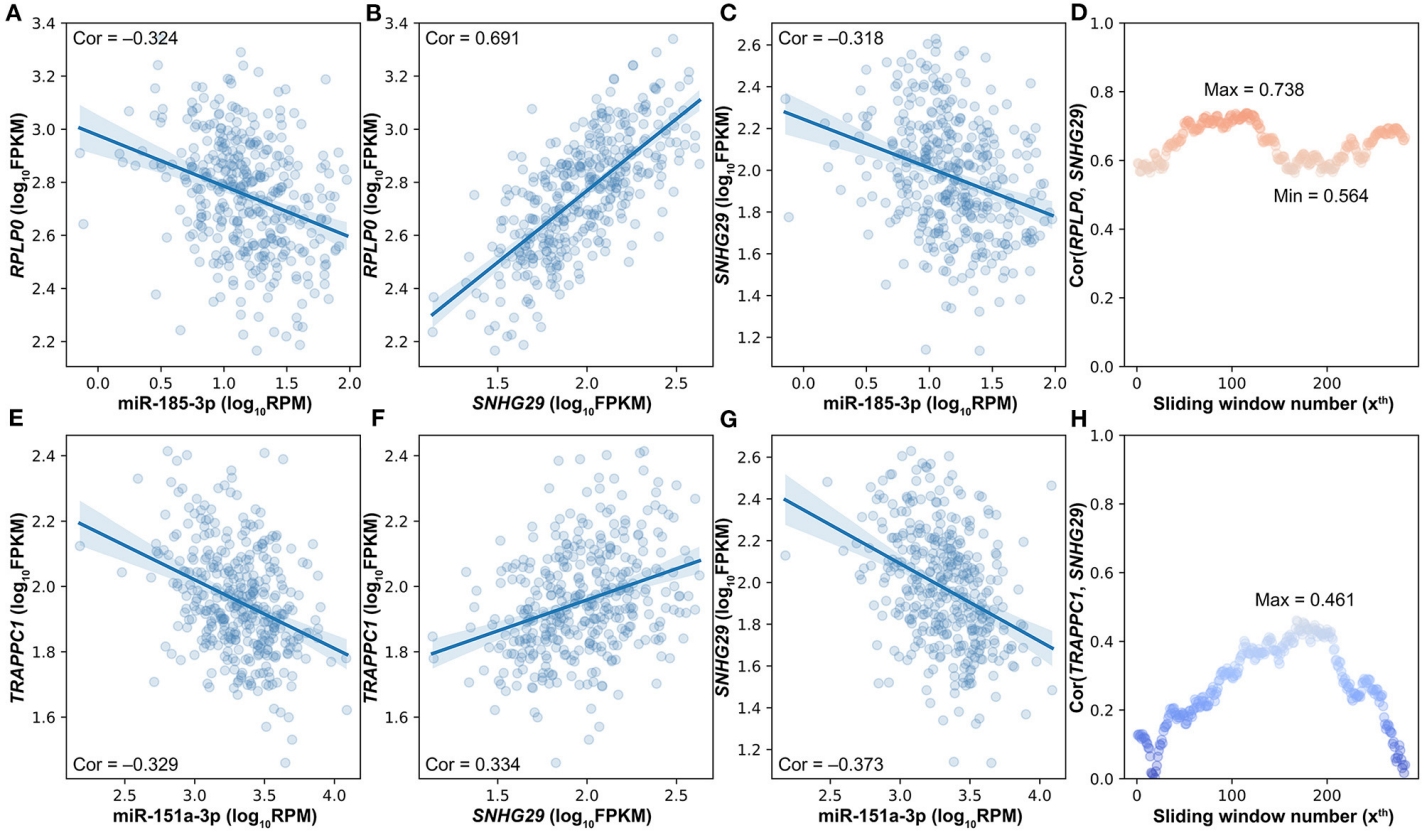
The impact of miRNA on the interaction between ceRNA pairs in triplets. **(A)** The scatter plot of miR-185-3p and *RPLP0*. **(B)** The scatter plot of SNHG29 and *RPLP0*. **(C)** The scatter plot of miR-185-3p and *SNHG29*. **(D)** The impact of miR-185-3p on the *SNHG29–RPLP0* interaction. **(E)** The scatter plot of miR-151a-3p and *TRAPPC1*. **(F)** The scatter plot of *SNHG29* and *TRAPPC1*. **(G)** The scatter plot of miR-151a-3p and *SNHG29*. **(H)** The impact of miR-151a-3p on the *SNHG29–TRAPPC1* interaction.

With the strictly selected negatively and positively correlated interactions, 256 competing triplets can be found by using the traditional strategy. If a miRNA is negatively correlated to two positively correlated target genes, then they form a competing triplet. As this traditional strategy ignores the mediating effect of miRNA on the positive relationship between target genes, several competing triplets may be fake ones. For example, miR-185-3p is negatively correlated to the two positively correlated target genes (Figures 3A–C); however, the positive correlation between SNHG29 and RPLP0 is not related to miR-185-3p (Figure 3D). According to the ceRNA hypothesis, SNHG29–miR-185-3p–RPLP0 is a fake competing triplet. Thus, the impact of miRNA on the interaction between ceRNA pairs should be considered.

To determine whether the interaction between target genes is derived from their relationship with miRNA, a commonly used method is to compare the correlation coefficients of target gene pairs under conditions of high and low miRNA expression levels. Accordingly, the differences of lncRNA–mRNA pairs' SCCs on the first and last quarter of samples sorted by miRNA expression were calculated, and 15 of the 256 competing triplets were identified to be true. A hidden hypothesis of this method is that the strength of the interaction between lncRNA and mRNA is linearly correlated with the miRNA expression level. However, according to the ceRNA hypothesis, both extremely high and extremely low miRNA expressions would impair the interaction between ceRNA pairs and even make them unrelated with each other. For example, miR-151a-3p is negatively correlated to the two positively correlated target genes (Figures 3E–G). The SCC between *TRAPPC1* and *SNHG29* is not linearly correlated with the expression level of miR-151a-3p (Figure 3H). The SCC achieves the maximum value at about the median miRNA expression level. Therefore, in LncMiM, all the miRNA expression levels, rather than only the highest and lowest ones, are considered when evaluating the impact of miRNA on the interaction between target gene pairs.

**Figure 4:**
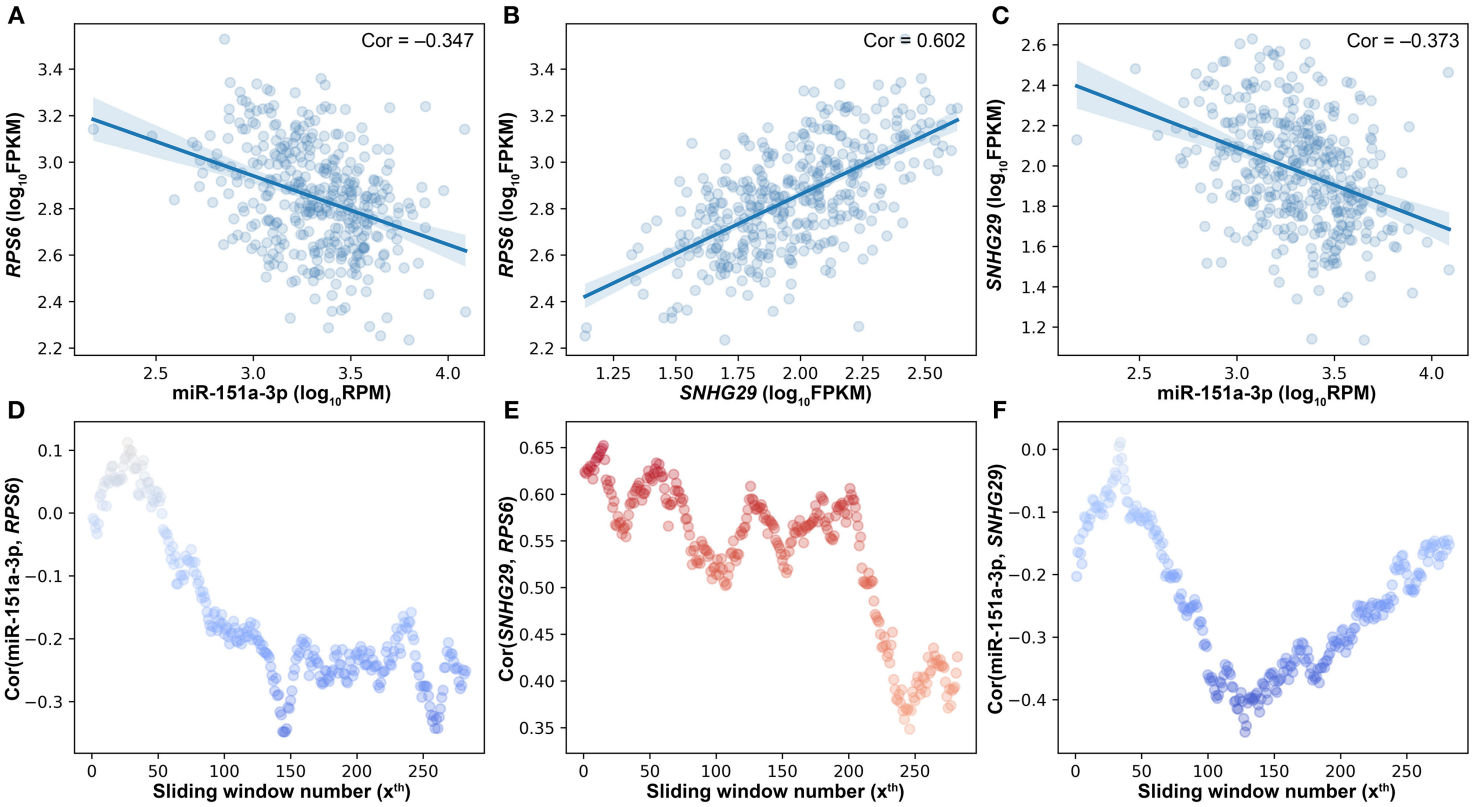
The pairwise interaction impacted by the other one in triplets. **(A)** The scatter plot of miR-151a-3p and *RPS6*. **(B)** The scatter plot of *SNHG29* and *RPS6*. **(C)** The scatter plot of miR-151a-3p and *SNHG29*. **(D)** The impact of *SNHG29* on the miR-151a-3p–*RPS6* interaction. **(E)** The impact of miR-151a-3p on the *SNHG29–RPS6* interaction. **(F)** The impact of *RPS6* on the miR-151a-3p–*SNHG29* interaction.

Besides the impact of miRNA on the lncRNA–mRNA interaction, lncRNA and mRNA can also affect the miRNA–target interactions. As shown in Figure 4, miR-151a-3p is negatively correlated to the two positively correlated target genes (*RPS6* and *SNHG29*). The SCC between *RPS6* and *SNHG29* is significantly changed with the rise of miR-151a-3p expression levels (Figure 4E). Moreover, the correlation relationship between *RPS6* and miR-151a-3p is impacted by the *SNHG29* (Figure 4D), and the interaction between *SNHG29* and miR-151a-3p is influenced by the *RPS6* (Figure 4F). As the pairwise interactions are impacted by the other one in the triplets, it is not enough to assess the real relationship between each pair only based on their own expression profiles, especially when the sample size is very large. The triplet with two correlated pairs may also be a competing triplet; thus, three types of candidate triplets were analyzed in LncMiM. Using the selected miRNA–target and lncRNA–mRNA pairs, 2060 “miRNA-centered” triplets, 1944 “lncRNA-centered” triplets, and 1537 “mRNA-centered” triplets were assembled. By using LncMiM, 231 “miRNA-centered” triplets, 339 “lncRNA-centered” triplets, and 439 “mRNA-centered” triplets were identified as competing triplets (Supplementary Table 1). In total, 847 competing triplets were found, including 38 miRNAs, 36 lncRNAs, and 236 mRNAs.

### Functional Analysis of the lncRNA-Associated Competing Triplets in Ovarian Cancer

In the competing triplets, a considerable number of lncRNAs, miRNAs, and mRNAs have been reported to be associated with ovarian cancer. By searching related literature and databases, about 30% lncRNAs have been verified to play roles in the regulation of proliferation, invasion, and migration of ovarian cancer cells, including *ZFAS1, SNHG1, GAS5, EMX20S, GIHCG, TP53TG1, EPB41L4A-AS1, SNHG8, SNHG6*, and *HCP5* (Zhan et al., 2018; Gao et al., 2019; Wu et al., 2019; Miao et al., 2020; Wang et al., 2020). In addition, some lncRNAs (e.g., *SNHG29, FGD5-AS1, TRIM52-AS1, EPB41L4A-AS1, RNASEH1-AS1, SNHG7, SPINT1-AS1, MAPKAPK5-AS1*, and *PITPNA-AS1*) are reported to be involved in other types of cancers (Wang et al., 2018; Gao et al., 2019, 2; Han et al., 2019; Zhou et al., 2020). Through retrieving the miRCancer database (version june2020) (Xie et al., 2013), 60.5% miRNAs in the competing triplets have been proved to be associated with ovarian cancer. In the mRNAs, 29 ovarian cancer oncogenes were found by searching the OCGene database (Liu et al., 2015). These results indicate that the lncRNA-associated competing triplets play important roles in the progression of ovarian cancer.

**Figure 5:**
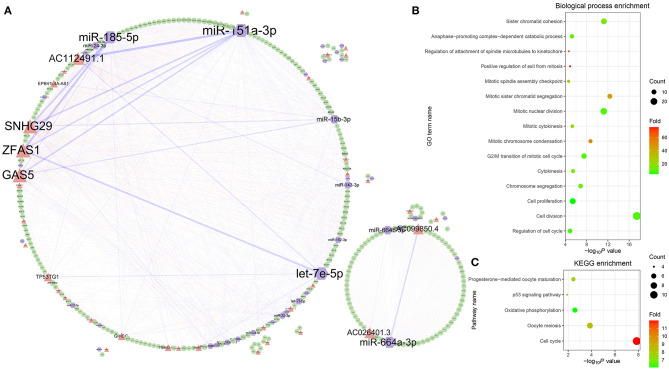
Comprehensive analysis of the lncRNA-associated competing triplets. **(A)** The regulatory network consists of all the competing triplets. **(B)** GO biological process enrichment analysis. **(C)** KEGG pathway enrichment analysis.

To analyze the regulatory relationship between lncRNA, miRNA, and mRNA in ovarian cancer, a comprehensive network was established through combining the 847 lncRNA-associated competing triplets (Figure 5A). In the network, 310 nodes are connected by 1,182 edges, including 132 miRNA–lncRNA edges, 539 miRNA–mRNA edges, and 511 lncRNA–mRNA edges. Among them, the top 10 nodes with most connections are miR-151a-3p, *ZFAS1, SNHG29*, miR-185-5p, *GAS5, AC112491.1*, let-7e-5p, miR-664a-3p, *AC099850.4*, and miR-15b-3p. The top 10 edges connected with most nodes are miR-151a-3p–*AC112491.1*, miR-185-5p–*ZFAS1*, miR-185-5p–*SNHG29*, miR-151a-3p–*GAS5*, let-7e-5p–*ZFAS1*, miR-664a-3p–*AC026401.3*, miR-151a-3p–*SNHG29*, miR-151a-3p–*ZFAS1*, miR-664b-3p–*AC099850.4*, and miR-15b-3p–*GAS5*. Based on the connections, the nodes are divided into two groups. The small group is mainly regulated by the miR-664a-3p and *AC026401.3* pair, while the ribosome protein-related mRNAs are all located in the large group.

Among the mRNAs, there are 39 *RPL* and 27 *RPS* genes, which indicates that the triplets are involved in the ribosome biogenesis. Except the *RP*s, the GO biological process enrichment analysis of the other genes shows that the competing triplets are also involved in cell division, cell proliferation, regulation of cell cycle, anaphase-promoting complex-dependent catabolic process, cytokinesis, chromosome segregation, and other nine processes related with cell mitosis (Figure 5B). In addition, the competing triplets are found to be mainly enriched in five KEGG pathways, including cell cycle, oocyte meiosis, oxidative phosphorylation, p53 signaling pathway, and progesterone-mediated oocyte maturation (Figure 5C). All the results suggest that the lncRNA-associated competing triplets mediate ovarian cancer progression through regulating ribosome biogenesis, cell cycle, cell division, and cell proliferation, and they may be associated with survival in patients with high-grade serous ovarian cancer.

### Identification of Potential Prognostic Competing Triplets

The Cox PH analysis was used to identify survival time associated miRNAs, mRNAs, and lncRNAs in the competing triplets. The result of univariate Cox PH analysis indicates that the lncRNA *FGD5-AS1* (*p* = 0.0008) is a potential prognostic biomarker for all patients with ovarian cancer. For patients in grade G2, *C12orf45, NDUFB8, POLR2J, SNRPE*, and *SNRPF* are found to be associated with survival time (*p* < 0.001). By multivariate analysis with patient age at diagnosis, more potential prognostic biomarkers are found, including *FGD5-AS1, GABPA, MRPS27, NR1D2*, and *NR2C2*. For patients in grade G2, only *SNRPF* is related to the survival time with the diagnosis age. For patients in grade G3, *FGD5-AS1, LETMD1, MAPKAPK5-AS1, MRPS27*, and *SDHC* are screened out as prognostic biomarkers with the diagnosis age. FGD5-AS1 and MRPS27 are found to be associated with the survival time of patients in stage IIIC, while *B9D1, RNASEH1-AS1, SPINT1-AS1*, and *ZWINT* are associated with the survival time of patients in stage IV. The association between the survival time and the triplet was also evaluated by using multivariate Cox PH analysis. With the threshold *p* < 0.001, miR-224-5p/*AL354892.2*/*ZBTB12* is found to be survival associated competing triplets. Considering the age of the patient at the initial pathologic diagnosis, 18 competing triplets are found to be associated with the overall survival time of patients in ovarian cancer, including miR-224-5p/*AL354892.2*/*ZBTB12*, miR-3653-3p/*FGD5-AS1*/*NR1D2*, miR-224-5p/*AC112491.1*/*NDUFB8*, and so on (Supplementary Table 2).

**Figure 6:**
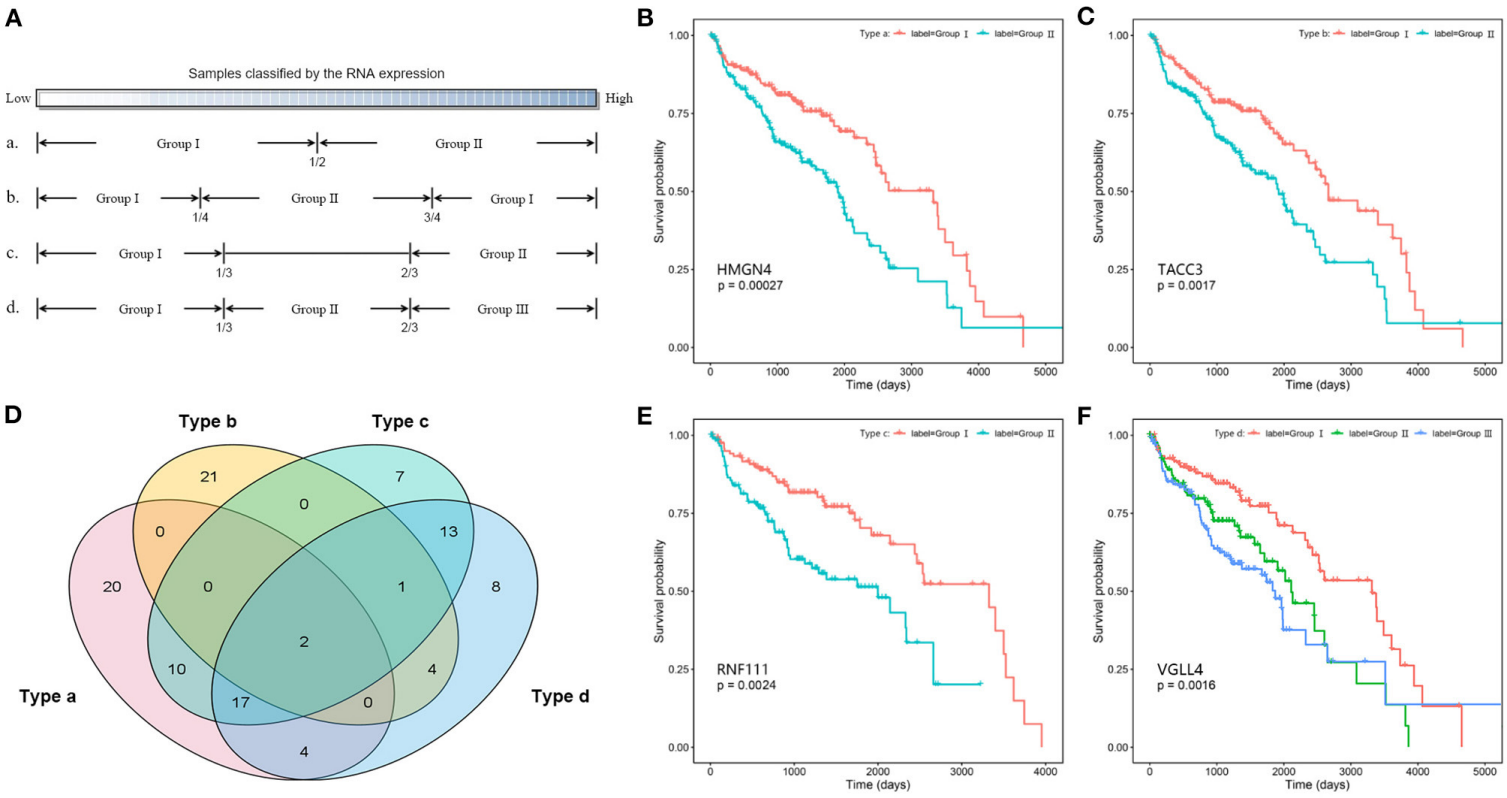
Survival analysis of competing triplets. **(A)** Four grouping modes. **(B)** The Kaplan–Meier curve of HMGN4 with mode a. **(C)** The Kaplan–Meier curve of TACC3 with mode b. **(D)** The Venn diagram of survival associated genes with four grouping modes. **(E)** The Kaplan–Meier curve of RNF111 with mode c. **(F)** The Kaplan–Meier curve of VGLL4 with mode d.

In addition, the Kaplan–Meier survival analysis was also performed to evaluate the potential prognostic power of miRNAs, lncRNAs, and mRNAs in the competing triplets. Considering the large data size, for each gene, the tumor samples were divided into two or three groups according to their expression levels by four ways (Figure 6A). By different grouping modes, a total of 107 RNAs are found to be associated with survival time, including 13 miRNAs, 10 lncRNAs, and 84 mRNAs (Supplementary Table 3). As show in Figure 6B, each grouping mode has its unique results. Especially, the grouping mode b has the least common results with the other modes, which indicates that there is a more complicated relationship between the patient survival time and the gene expression value. For each grouping mode, the most significant genes are *HMGN4, TACC3, RNF111*, and *VGLL4* (Figures 6B,C,E,F). The survival associated genes are involved in 368 competing triplets, which are found to be enriched in cell division, cell proliferation, ribosome, and cell cycle.

## Discussion

In this study, TargetScan, PITA, miRanda, and miRTarBase were used together to predict miRNA–target pairs, and a total of 2,608,237 miRNA–mRNA and 74,086 miRNA–lncRNA interactions were found (Supplementary Table 4). As shown in Figures 2A,D, each tool exclusively predicted a fraction of miRNA–target interactions. Although a vast number of miRNA–target interactions were predicted by TargetScan, PITA, and miRanda, there are still several experimentally validated miRNA–target interactions predicted by none of these tools. miRNA–mRNA pairs together with miRNA–lncRNA pairs could construct a huge number of triplets (~1.69E+9). Considering the computation and time cost, miRNA–target pairs were firstly filtered by correlation relationships.

Through the miRNA–target relationship, 1,816,605 indirect interactions between mRNA and lncRNA were established. Based on the linear relationship calculated by the PCC, 3 negatively correlated miRNA–lncRNA pairs (*PCC* = −0.3), 443 negatively correlated miRNA–mRNA pairs (*PCC* = −0.3), and 27,897 positively correlated lncRNA–mRNA pairs (*PCC* > 0.3) were screened out. With the linearly correlated pairs, 64 competing triplets were established. The impact of miRNA on the linear relationship between lncRNA and mRNA was only found in seven competing triplets. In contrast, based on the non-linear relationship assessed by the SCC, 89 negatively correlated miRNA–lncRNA pairs (*SCC* = −0.3), 3,586 negatively correlated miRNA–mRNA pairs (*SCC* < −0.3), and 33,267 positively correlated lncRNA–mRNA pairs (*SCC* > 0.3) were screened out. Comparing with the PCC, more negatively correlated miRNA–target pairs are found by the SCC.

In most of the scatter plots of the negatively correlated miRNA–target pairs, the points are mainly located in the bottom left corner, which looks like a triangle other than a line (Figure 2E). By comparison, after normalizing expression values by a logarithmic transformation, the points become more dispersed and scatter around a line. This result indicates that the linear correlation between miRNA and the target is impacted by the large span of the expression values, which is brought by the large sample size. In addition, the different orders of magnitude of the expression value between miRNA and the target gene are also an impact factor. The expression value of miRNA is calculated by RPM (max value: 8.23E5), while the expression values of mRNA and lncRNA are calculated by FPKM (max value of mRNA: 2.15E4, max value of lncRNA: 1.85E3). Therefore, it is better to assess the relationship between miRNA and the target by the non-linear correlation, especially on the large scale of data.

The bigger the size of the patient data, the more complex the relationships between ceRNAs we can observe. According to the ceRNA hypothesis, the strength of the competing relationship between ceRNAs is not constant but depends on the amount of miRNA (Figures 3H, 4E). Similarly, the strength of the interaction between miRNA and ceRNA is also impacted by the amount of the other ceRNA (Figures 4D,F). In 231 competing triplets, miRNAs are negatively correlated to the mRNAs and lncRNAs. Although the positive correlation between mRNA and lncRNA is not significant on the whole samples, their correlation is changed with the expression level of miRNA, and a significant positive correlation can be observed on a specific subset of samples. In 778 competing triplets, the negative correlation between miRNA and ceRNA is not significant on the whole samples, but there is a significant negative correlation on a specific subset of samples, and the correlation is influenced by the other ceRNA. Thus, besides the impact of miRNA on the interaction between ceRNAs, the impact of ceRNA on the correlation between miRNA and other ceRNAs should also be considered.

However, there is still no method considering the impact of both the miRNA and the ceRNAs when identifying competing triplets. The method, sensitivity partial Pearson correlation (SPPC), only estimates the impact (sensitivity) of miRNA on the interactions between ceRNAs (Paci et al., 2014). However, when using SPPC on “miRNA-centered” candidate triplets, no competing triplets were identified. JAMI is a conditional mutual information-based method, which can only estimate the impact of ceRNA on the interaction between miRNA and other ceRNAs (Hornakova et al., 2018). With JAMI, 87 competing triplets were filtered out from 1,507 “mRNA-centered” candidate triplets, and 385 competing triplets were identified from 1,944 “lncRNA-centered” candidate triplets. The JAMI results only show that the centered ceRNA has a significant influence on the relationship between miRNA and the other RNA in a candidate triplet, but it is still unknown if the other RNA is a ceRNA that should be negatively correlated with miRNA. In addition, the *SNHG29*/miR-151a-3p/*RPS6* competing triplet is not identified by JAMI (Figure 4).

Considering the drawbacks of the existing tool, we developed a novel method named LncMiM to identify lncRNA-associated competing triplets in ovarian cancer. Besides the impact of miRNA on the interaction between ceRNA pairs, the impact of ceRNA on the interaction between miRNA and the other ceRNA is also used to identify competing triplets. As compared with other tools, LncMiM shows better performance (Supplementary Table 5). By using LncMiM, 231 competing triplets were identified from 2,060 “miRNA-centered” candidate triplets, 339 competing triplets were identified from 1,944 “lncRNA-centered” triplets, and 439 competing triplets were identified from 1,507 “mRNA-centered” triplets. In final, a total of 847 lncRNA-associated competing triplets were found. The functional enrichment analysis shows that the competing triplets are mainly involved in cell division, cell proliferation, and regulation of cell cycle. The KEGG pathway analysis shows that they are associated with ribosome, cell cycle, oocyte meiosis, oxidative phosphorylation, p53 signaling pathway, and progesterone-mediated oocyte maturation. Among them, 18 competing triplets are found to be significantly correlated with the overall survival in ovarian cancer.

## Data Availability Statement

The original contributions presented in the study are included in the article/Supplementary Materials, further inquiries can be directed to the corresponding authors.

## Author Contributions

JZ developed the LncMiM method, identified competing triplets, and wrote the manuscript. TX and QY collected and calculated the RNA expression profiles of ovarian cancer. JL and BJ identified the potential prognostic triplets. JW and XS conceived the study, supervised the work, manuscript writing, and editing. All authors read and approved the final manuscript.

## Conflict of Interest

The authors declare that the research was conducted in the absence of any commercial or financial relationships that could be construed as a potential conflict of interest.
