# Supplementary material for: Identification of Potential Prognostic Competing Triplets in High-Grade Serous Ovarian Cancer
Source: Front Genet. 2021 Jan 13;11:607722. doi: 10.3389/fgene.2020.607722 (PMC7839966; doi:10.3389/fgene.2020.607722)
Supplement: Supplementary file 1 [file Data_Sheet_1.zip › Tables 4- 5.docx]

# Supplementary material

Table S1. The list of lncRNA-competing triplets.

Table S2. The results of survival analysis.

Table S3. The list of competing triplets associated with the overall survival in ovarian cancer.

Table S4. The results of miRNA-target pairs derived from TargetScan, miRanda, PITA, and miRTarBase.

Table S5. Comparison with other existing tools for identifying competing triplets in ovarian cancer.

Table S4. The results of miRNA-target pairs derived from TargetScan, miRanda, PITA, and miRTarBase.

|  | TargetScan | miRanda | PITA | miRTarBase | Total |
| --- | --- | --- | --- | --- | --- |
| miRNA-mRNA | 511,131 | 2,305,301 | 2,436,470 | 51,197 | 2,608,237 |
| miRNA-lncRNA | 20,573 | 66,177 | 69,288 | 26 | 74,086 |
| Total | 531,704 | 2,371,478 | 2,505,758 | 51,223 | 2,682,323 |

Table S5. Comparison with other existing methods for identifying competing triplets in ovarian cancer.

|  | PCC | SCC | SPPC | JAMI | LncMiM |
| --- | --- | --- | --- | --- | --- |
| miRNA-centered candidate triplets | 15 | 1166 | 0 | NA | 231 |
| lncRNA-centered candidate triplets | 2 | 283 | NA | 672 | 339 |
| mRNA-centered candidate triplets | 4 | 256 | NA | 145 | 439 |
| Total | 19 | 1578 | 0 | 809 | 847 |

Filtering criteria for other existing methods were set as follows. PCC and SCC: cor(miRNA,lncRNA) < –0.3, cor(miRNA,mRNA) < –0.3, cor(mRNA,lncRNA) > 0.3, and *p-value* < 0.05; SPPC: cor(lncRNA,mRNA) – cor(lncRNA,mRNA|miRNA) > 0.3; JAMI: adjusted *p-value* < 0.05.

SPPC only estimates the impact of miRNA on the interactions between ceRNAs. JAMI calculates the difference between CMI(miRNA, gene A | gene B) and MI(miRNA, gene A), then uses the adjusted p-value to screening competing triplets. JAMI cannot estimate the impact of miRNA on the interactions between ceRNAs. In addition, for JAMI, it requires that both the gene A and gene B are the miRNA target genes. However, for lncRNA- and mRNA-centered candidate triplets, the gene A may be not the miRNA target gene, thus it needs to further identify their regulatory relationship. Considering the regulatory relationship between miRNA and gene A is influenced by gene B, it is not easy to identify their relationship on the whole samples.

Due to the drawbacks of the PC- and CMI-based methods, we developed a novel method – LncMiM to identifying competing triplets. Comparing with SPPC and JAMI, LncMiM can both estimate the impact of miRNA on the interaction between ceRNA pairs and the impact of ceRNA on the interaction between miRNA and other ceRNAs. In addition, LncMiM can evaluate the regulatory relationship between miRNA and ceRNA with consideration of the expression level of the other ceRNA in the candidate triplets.
